# Reinforced Concrete Slabs Strengthened with Lap-Spliced Carbon TRC System

**DOI:** 10.3390/ma14123340

**Published:** 2021-06-17

**Authors:** Hyeong-Yeol Kim, Young-Jun You, Gum-Sung Ryu

**Affiliations:** Structural Engineering Department, Korea Institute of Civil Engineering and Building Technology (KICT), Goyang 10223, Korea; hykim1@kict.re.kr (H.-Y.K.); yjyou@kict.re.kr (Y.-J.Y.)

**Keywords:** carbon textile, textile-reinforced concrete (TRC), lap splice, grout, structural testing, flexural strengthening, tensile test

## Abstract

Construction with precast or prefabricated elements requires the connecting of structural joints. This study presents an accelerated construction method to strengthen reinforced concrete (RC) slab-type elements in flexure using precast lap-spliced textile-reinforced concrete (TRC) panels. The objectives of this study are to identify the tensile behavior of a TRC system with lap-spliced textile, and to experimentally validate the performance of the proposed connecting method by flexural failure test for the concrete slabs strengthened by TRC panels with lap-spliced textile. Twenty-one coupon specimens were tested in tension with two different matrix systems and three different lap splice lengths. The influence of the lap splice length and matrix properties on the tensile performance of the TRC system was significant. Five full-scale RC slabs were strengthened by the precast TRC panels with and without the lap splice, and was tested in flexure. The results of the failure test for the strengthened specimens showed that the ultimate load of the strengthened specimen with the TRC panel increased by a maximum of 24%, compared to that of the unstrengthened specimen. Moreover, the failure-tested specimens were re-strengthened by a new TRC panel system and tested again in flexure. The objective of the re-strengthening of the damaged RC slabs by the TRC panel is to investigate whether the yielded steel reinforcement can be replaced by the TRC panel. The initial cracking load and the stiffness of the re-strengthened specimens were significantly increased by re-strengthening.

## 1. Introduction

**Figure 1:**
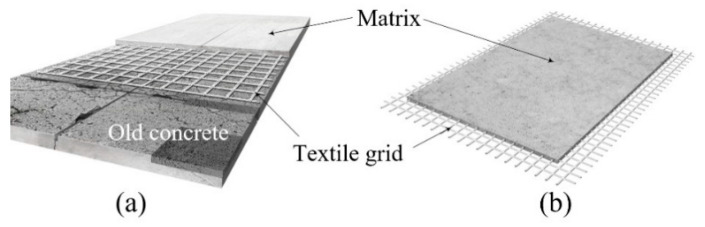
Schematics of TRC system used for: (**a**) repair or strengthening; and (**b**) precast element.

Textile-reinforced concrete (TRC) is composed of textile reinforcement and a matrix and has a much thinner layer than conventional reinforced concrete (RC). The textile reinforcement generally takes the form of a two-dimensional (2D) grid of carbon fibers, and the matrix is commonly made of concrete or cementitious mortar. If the matrix contains no coarse aggregates, we refer to it as textile reinforced mortar (TRM). TRC is more popularly used than TRM. The TRC system can be fabricated either by on-site cast-in-place (CIP) installation or off-site precast element. Figure 1a,b respectively illustrate a TRC system installed over an existing concrete element and a precast TRC element.

Over the past two decades, the TRC system has been widely studied for both construction of new structural components and strengthening of existing structures. Early developments are well summarised in the literature [1,2,3,4], and recent developments and applications of the TRC system can be found in References. [5,6,7,8,9,10,11,12,13]. More recently, a research group in the Korea Institute of Civil Engineering and Building Technology (KICT) conducted a series of experimental investigations to utilise a TRC system for a new structural component and strengthening for deteriorated concrete elements. In KICT, slab-type concrete elements were strengthened with a precast TRC panel with grout [14], as well as a CIP TRC system [15,16]. A precast TRC panel was also used for stay-in-place permanent formwork in the new construction of a reinforced concrete slab [17]. Moreover, the long-term tensile performance of the TRC system was investigated through collaboration by research groups in KICT and ITA RWTH Aachen University (Aachen, Germany) [18].

In earlier applications, lack of testing methods and design guidelines for TRC systems was one of the technical barriers to enter construction markets. However, the testing methods and design guidelines for TRC systems in building and construction have been relatively slow, but successfully established in recent years [19,20,21,22]. Although roughly specified in the design guidelines for TRC systems [22], designers dealing with the TRC system must pay attention to overlap or lap splice length of the textile reinforcements. Every commercially available 2D textile grid reinforcement is fabricated either by a warp-weaving or warp-knitting method, where the knitting method is more popular these days. Although technically there is no limitation of textile fabrication in length, e.g., warp direction, the width of textile is generally limited by the machine size. Therefore, the textile grid often requires on-site overlap in the weft direction of the textile.

The overlapped textile grid has a mechanical discontinuity, and is a weak point for the TRC system. The induced tensile stress within the TRC system should be transferred from one textile grid to another through the matrix. To safely transfer tensile stress of the lap-spliced textile, the overlap length (hereafter, lap splice length) of the textile grid must be greater than the minimum lap splice length of the textile grid. The minimum lap splice length of the textile grid is usually evaluated by a tensile test in accordance with a testing protocol, such as that specified in [22]. Numerous research groups [23,24,25] conducted direct tensile tests for TRC coupon specimens fabricated with lap-spliced textile grids. Overall, longer lap-spliced length of the textile grid gave better tensile performance than the shorter length.

**Figure 2:**
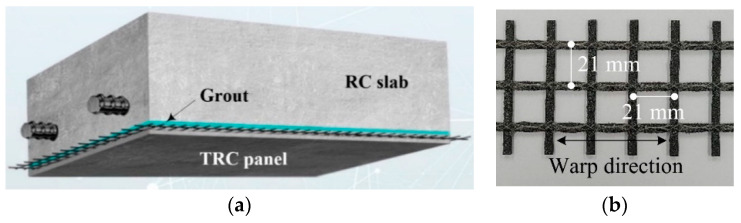
(**a**) Schematics of strengthening of concrete element with TRC panel and grout; and (**b**) carbon textile grid (Anticrack Q85/85-CCE-21-E4, Solidian-Kelteks Co. Ltd. Karlovac, Croatia).

The strengthening of concrete structures with TRC system is usually carried out on-site, but on-site casting methods need to be modified for sites with a narrow working space. A fast installation of TRC system can be obtained if a precast TRC panel can be externally bonded to an existing concrete structure, as presented by KICT [14,26,27,28]. As illustrated in Figure 2, the TRC panel can be installed underneath concrete elements by anchor bolts with a grouting space between the panel and the concrete. Several air vents and grouting inlets are also installed. The grout is then injected until the grout completely fills the space between the panel and the concrete. A preceding paper [14] reported the development and validation of an externally bonding method using a precast TRC panel and cementitious grout for the strengthening of concrete elements, such as the slab deck of an open-type wharf structure, and its effectiveness was demonstrated by a trial construction [26,27,28]. Note that the bottom reinforcement of slab deck of an open-type wharf structure is usually severely corroded due to chloride attack, if steel corrosion is not prevented, but the bottom surface of the deck is extremely difficult to repair or strengthen due to high water levels and narrow working space. Furthermore, an off-site method of repair or strengthening methods are highly recommended for these type of structures to avoid marine pollution during on-site construction. However, construction with precast or prefabricated elements always involves unavoidable structural joints, and requires connecting methods for the joints. Therefore, as suggested in the preceding study [14], an effective connecting method for the precast TRC panel joints should be developed for the externally bonded strengthening method by precast TRC panels and cementitious grout.

This paper deals with the development of an on-site connecting method for precast TRC panels with a lap-spliced textile grid. The objectives of this study are to identify the tensile behavior of the TRC system with lap-spliced textiles and to experimentally validate the performance of the proposed connecting method by flexural failure tests for concrete slabs strengthened by TRC panels with lap-spliced textile.

To examine the tensile behavior of a TRC system with lap-spliced textiles, prismatic TRC coupon specimens with lap-spliced carbon textile grids at the middle of the specimens were fabricated and tested under tension. Two different cementitious binders, e.g., mortar and non-shrink grout, were considered for the matrix system. Three different lap splice lengths were considered for the TRC system with mortar, while two different lap splice lengths were considered for the TRC system with grout. Twenty-one coupon specimens were tested in tension. Tensile properties of the lap-spliced specimens compared with those of the specimen without lap splice.

Six 2000.0 mm-long full-scale RC concrete slabs were fabricated and TRC panels (20.0 mm-thick) with one ply of carbon grid textile and mortar were also fabricated: one full-length (1600.0 mm) panel has no lap splice; four sets of the panels have a textile lap splice at the middle of the panel. Five RC slabs were strengthened with precast TRC panels and grout. Among these, one of the slabs was strengthened by a full-length TRC panel (no lap splice), two of the slabs were strengthened by a set of lap-spliced TRC panels at the mid span only and the other two slabs were strengthened by lap-spliced TRC panels with an additional textile grid within the grouting space to reinforced the joint. The full-scale strengthened slab specimens were tested by a three-point bending test. The load-deflection behavior and failure mode of the strengthened specimens is compared with those of the unstrengthened RC slab specimen.

## 2. Tensile Behavior of TRC System with Lap Splice

### 2.1. Materials

The carbon textile grid (Figure 2b) is employed in this study. Table 1 summarises the mechanical properties of the grid, which is sand-coated (grain size of 0.3–0.8 mm) [29]. The sand-coated textile is employed in this study, as the surface coated textiles showed better bonding performance than uncoated textiles [15,16,17,23,24,25].

The mix composition of the mortar [16] is summarised in Table 2. PVA (polyvinyl alcohol, KURALON K-II REC100L, Kuraray, Japan) short fibers (10%, length = 6.0 mm) was mixed with the mortar. Detailed explanations of the purpose and the process of the mix design of mortar for the TRC panel can be found in a previous paper [16]. The mean compressive strength of the air-cured mortar (ten cubic samples) at the age of 28 days was 63.9 MPa, with a standard deviation of 6.7.

On the other hand, a commercially available cementitious non-shrink grout (MR5000, Jetcon Ltd. Co., Seoul, Korea) was used as a grouting material in the fabrication of the specimens. The mean compressive strengths of the air-cured grout (ten cubic samples) at the time of the test was 81.6 MPa, with a standard deviation of 6.6 at the age of 28 days.

Furthermore, a ready-mixed concrete with a design strength of 27 MPa was used for the fabrication of full-scale RC slabs. The mix composition of the ready-mixed concrete is provided in Table 3.

### 2.2. Fabrication of Tensile Test Specimens

**Figure 3:**
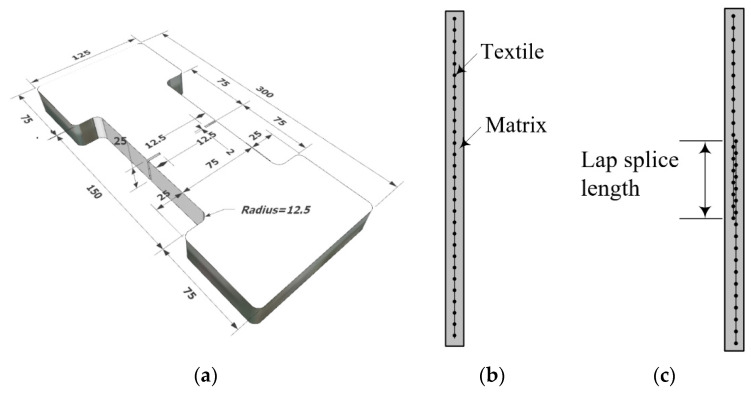
Coupon specimen for direct tensile test: (**a**) Dimensions (units, mm); (**b**) coupon without lap splice; and (**c**) coupon with a lap splice.

In this study, a direct tensile test was used to evaluate the tensile properties of the TRC system. Preliminary tests conducted in KICT indicate that the dumbbell-type coupon specimen (Figure 3a) provided more consistent test results than the clevis-type specimen specified in [20,22]. Two 12.5 mm-deep notches were provided at the middle of the specimen to induce a crack in this region. Figure 3b,c respectively show a side view of the specimen without a textile lap splice and with a lap splice.

Table 4 summarises the characteristics of two groups of coupon specimens tested under tension. The design variables considered in the test were type of matrix and lap splice length ($L_{LS}$). The first group of specimens (TM series) used a cementitious mortar, while the second group of specimens (TG series) used a cementitious grout. The maximum $L_{LS}$ was set to 150 mm by considering the length of the load transition zone of the coupon specimen, e.g., 150 mm. For both groups, textiles with no lap splice (TM-0 and TG-0 series) and with different lap splice length ($L_{LS}$) (0 mm–150 mm) were considered. Note that AC434 codes [22] recommends the minimum $L_{LS}$ of textile as 51 mm.

**Figure 4:**
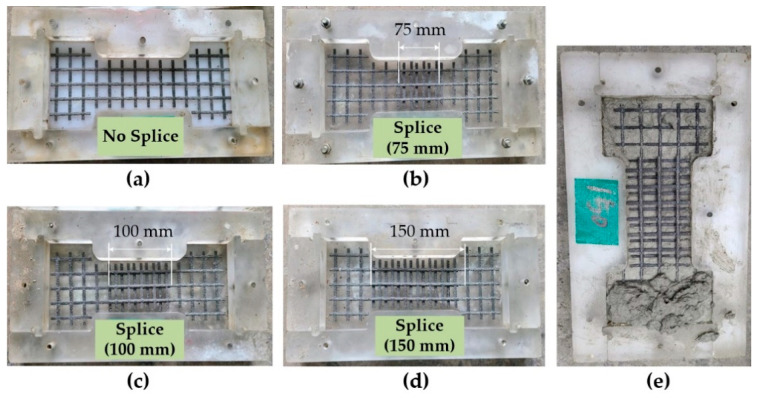
Textile grid placement for specimens with mortar (TM series): (**a**) no splice; (**b**) 75 mm lap splice; (**c**) 100 mm lap splice; (**d**) 150 mm lap splice; and (**e**) mortar placement (TM-150).

Figure 4a–d show the placement of textile within a plastic mold for the fabrication of the TM series coupon specimens. As illustrated in Figure 4e, the lap-spliced textile grid was placed mid-plane of the mortar layer.

**Figure 5:**
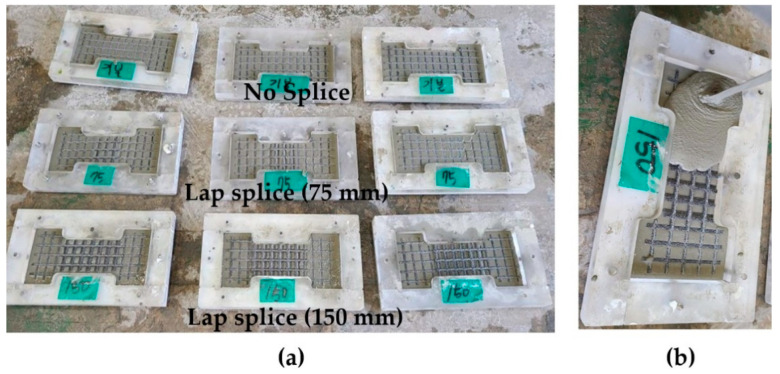
(**a**) Textile grid placement for TG series specimens with grout; and (**b**) grouting (TG-150).

Figure 5a,b respectively illustrate the placement of textile and the grouting within a mold for the fabrication of the TG series coupon specimens. First, the grout was poured onto the mold to form a half the thickness of the specimen (Figure 5b). The carbon textile was placed onto the first layer of the grout surface, and then the top surface of the specimen was finished by grouting.

### 2.3. Results of Tensile Tests and Discussion

**Figure 6:**
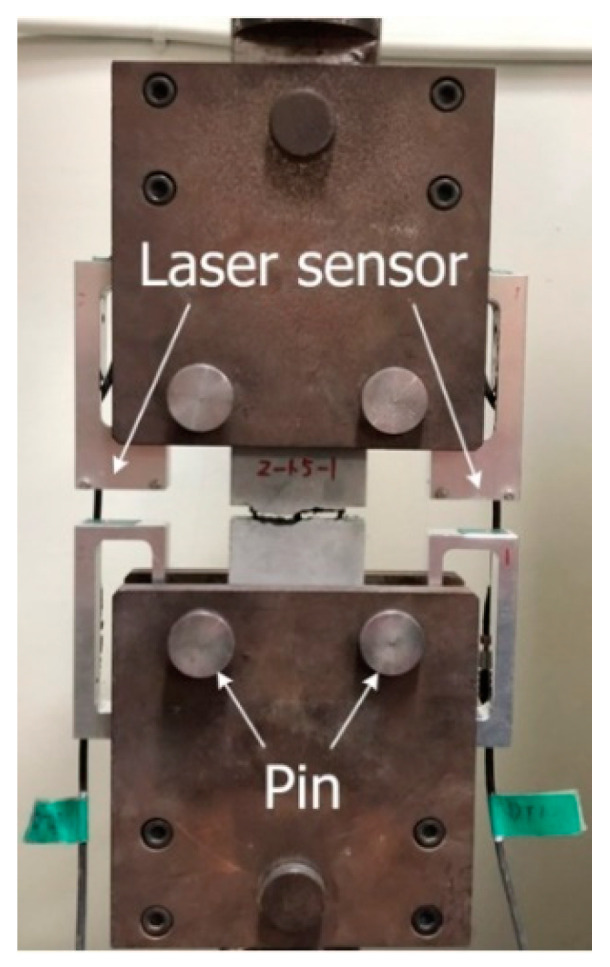
Set-up and instrumentation for direct tensile test.

Figure 6 shows the set-up for the direct tensile test. Vertical monotonic loading with a displacement control of 0.4 mm/min was applied to the specimens using a 300 kN capacity universal testing machine. Laser sensors mounted at both sides of the specimen measured the vertical displacement of the specimens.

**Figure 7:**
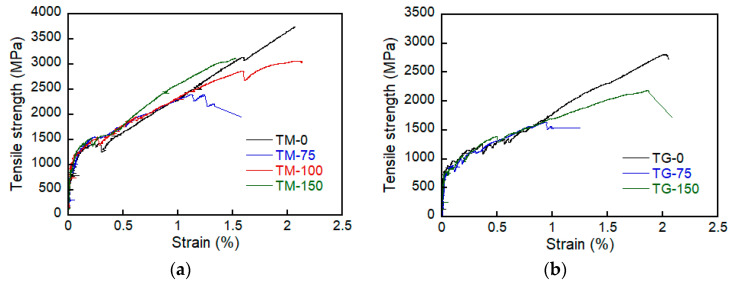
Axial stress-strain curve of specimens. (**a**) TM series; and (**b**) TG series.

The results of the direct tensile tests were summarised in Table 5. Figure 7 shows the axial stress-strain curve of the specimens. The ultimate tensile strength ($f_{fu}$) of the TRC system was significantly affected by the lap splice lengths. In the early stage of loading, the stress increased linearly until a matrix crack occurred. The sudden stress drop after matrix cracking is due to a local slip between the textile and matrix. The slope of the stress-strain curve then decreased, but the stress continued to increase until failure occurred.

Overall, $f_{fu}$ of the TM series specimens increased as $L_{LS}$ increased. However, $f_{fu}$ did not increase further for the lap splice length that was greater than 100 mm. Therefore, the minimum $L_{LS}$ of the TM series specimens can be considered as 100 mm. On the other hand, the initial cracking strength ($f_{cr}$) and the ultimate tensile strain ($\varepsilon_{fu}$) did not give consistent results with respect to $L_{LS}$. Similar behavior was also observed for the TG series specimens, but the testing variable ($L_{LS}$) was not sufficient to clearly identify the minimum $L_{LS}$. The maximum value of $f_{fu}$ of the TM and TG series specimens with the lap-spliced panel is at most 84.6% and 78.6% of the specimen with the full-length panel (no lap splice).

Although the compressive strength of the mortar (TM series) is about 78% of that of the grout (TG series), for the same value of $L_{LS}$, $f_{fu}$ of the TM series specimens was almost two times greater than that of the TG series specimens. Furthermore, $f_{cr}$ of the TM series was at least 27% greater than that of the TG series. Note that $f_{cr}$ is the initial cracking load divided by the cross-sectional area of the matrix (transition zone). Therefore, the influence of the matrix properties on $f_{fu}$ and $f_{cr}$ is significant.

**Figure 8:**
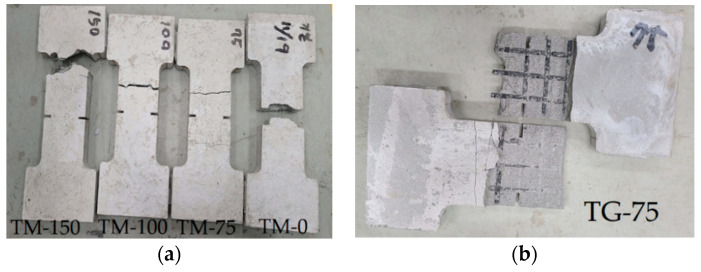
Failure modes of the tensile tested specimens. (**a**) TM series; (**b**) TG-75.

Figure 8 shows the failure modes of the tensile tested specimens. It can be clearly seen that failure of the TM-0 and TM-150 specimens is associated with tensile rupture of the textile grids, while failure of the TM-75 and TM-100 specimens is slippage of the textile within the matrix. On the other hand, failure of the TG series specimens is associated with splitting, and finally delamination along the lap-spliced face of the textile. As shown in Figure 8, few specimens were failed at 12.5 mm-deep notches that were placed at both sides of the coupons induce a crack in this region notches. Although an attempt was not made in this study, an analytical model in terms of stress intensity factor and fracture mechanics criteria needs to be developed to analyse the matrix cracking and design the notches.

The mortar used for the TM series specimens was reinforced with short dispersed PVA fibers. It should be further noted that the TM series specimens showed fewer stress drops than the TG specimens. Therefore, the results of direct tensile test indicated that the presence of PVA fibers in the mortar provided a crack-bridging force that prevented the TM series specimens from splitting failure.

To connect the joint of lap-spliced TRC panels on-site, grout is a preferred material over dense mortar. Therefore, a viable grouting material should be developed in a future study for the proposed method of on-site installation.

## 3. Flexural Behavior of Strengthened Slabs

### 3.1. Fabrication of Full-Scale Slab Specimens

**Figure 9:**
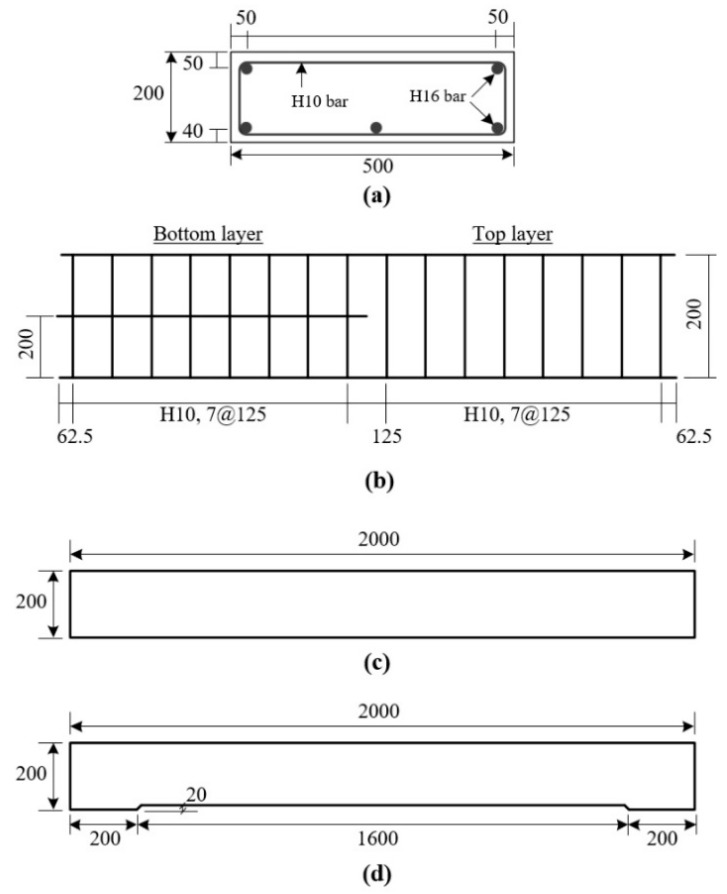
RC slab specimen: (**a**) cross-sectional dimensions; (**b**) reinforcement details; (**c**) side view of RC specimen; side view of RC slab to be strengthened with TRC panel; and (**d**) side view of RC slab with indented space.

Six 500 × 200 × 2000 mm^3^ (width × height × length) RC slabs were fabricated for a flexure failure test. Figure 9a,b respectively show arrangements of longitudinal reinforcing bars (H16, diameter = 15.9 mm) and stirrups (H10, diameter = 9.53 mm). As shown in Figure 9d, the RC slabs to be strengthened with the TRC panel were fabricated to have indented space (depth = 20 mm) underneath the bottom bars to fill the grouting material. The bottom face of the RC slab was assumed to be deteriorated, and the deteriorated section was chipped to the surface of the tensile reinforcement. The RC slabs were air-cured at 20 °C for 20 days.

**Figure 10:**
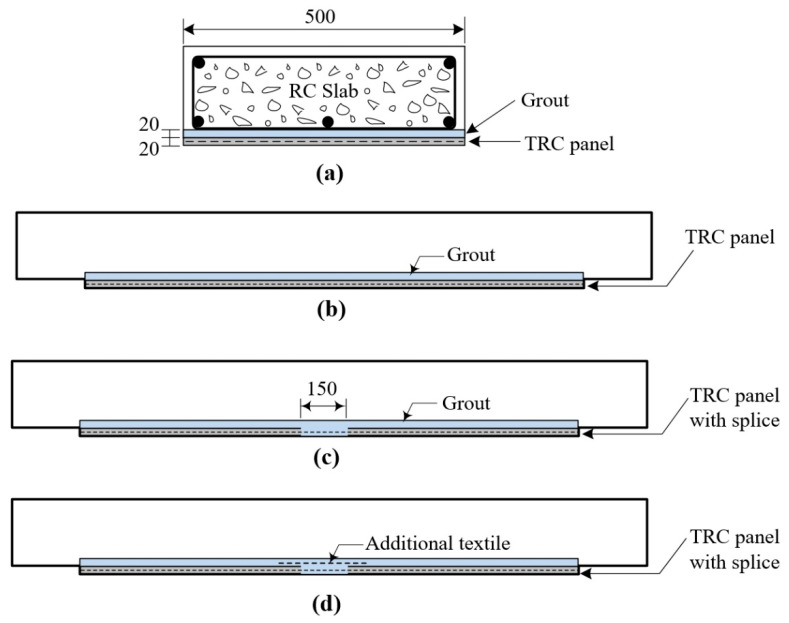
TRC system strengthening plan: (**a**) cross-sectional view; (**b**) TRC panel without splice; (**c**) TRC panel with a splice; and (**d**) placing additional textile at splice.

Table 6 lists characteristics of the six full-scale RC slab specimens fabricated for a flexure test. Figure 10 illustrates a series of strengthening plans for the RC slabs with the TRC panel (hereinafter the strengthened slabs). Among them, RC specimen is the control specimen, the S-1 specimen the RC slab strengthened with the full-length TRC panel and the S-2 and S-3 series specimens the RC slab strengthened with the lap-spliced TRC panel. The lap splice joint is located at the mid-span of the RC slabs. Note that the lap slice joint of the S-3 specimens was further reinforced with an additional 300 mm-long textile grid within the grouting space. The designed lap splice length is 150 mm, and the lap splice joint is completely filled with grout during the strengthening work.

### 3.2. Fabrication of TRC Panel

**Figure 11:**
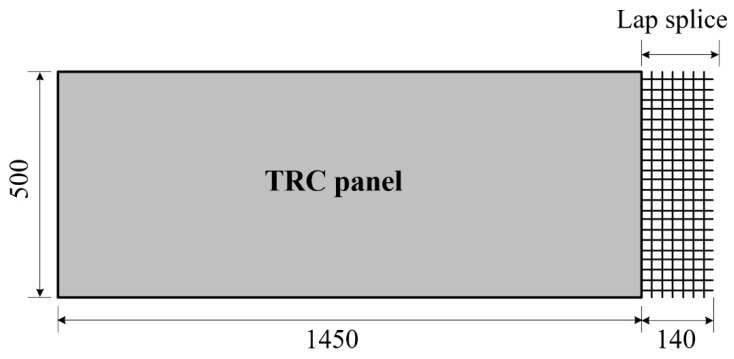
Precast lap-spliced TRC panel (unit: mm).

Precast TRC panels (500 mm wide and 20 mm thick) were fabricated with carbon textile grid (Table 1) and mortar (Table 2). In this study, two different types of TRC panels were fabricated: the first type is a full-length (1600 mm) panel without a lap splice, and the second is a lap-spliced panel (Figure 11). One side of the lap-spliced panel in the direction of the slab length has an extruded textile with a length of 140 mm.

**Figure 12:**
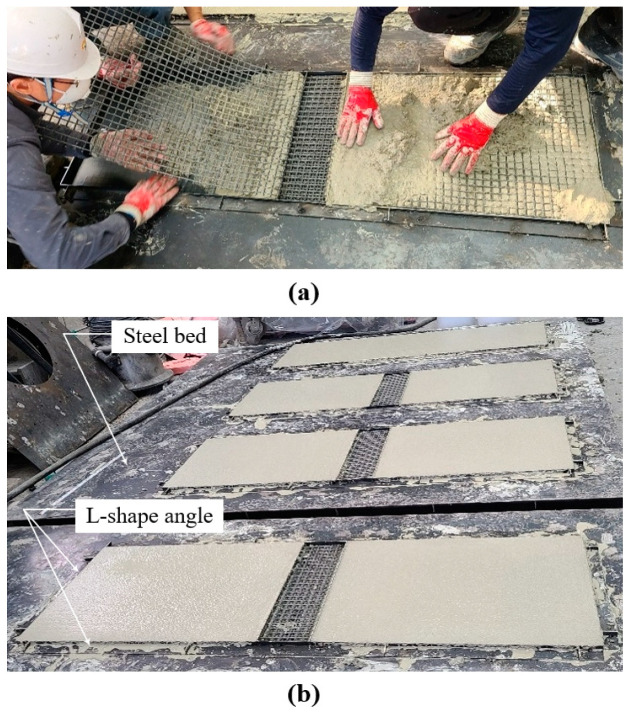
Fabrication of lap-spliced TRC panel: (**a**) installation of textile grid; and (**b**) the placing of mortar.

Figure 12 illustrates the fabrication process of the lap-spliced TRC panel. Mortar was not placed in the lap splice joint (Figure 12a). A set of the textile grids was then placed with a lap slice length of 150 mm. The remaining thickness of mortar was then placed and finished. Figure 12b show both types of panels after being finished by a trowel.

### 3.3. Strengthening of RC Slab with TRC Panel

**Figure 13:**
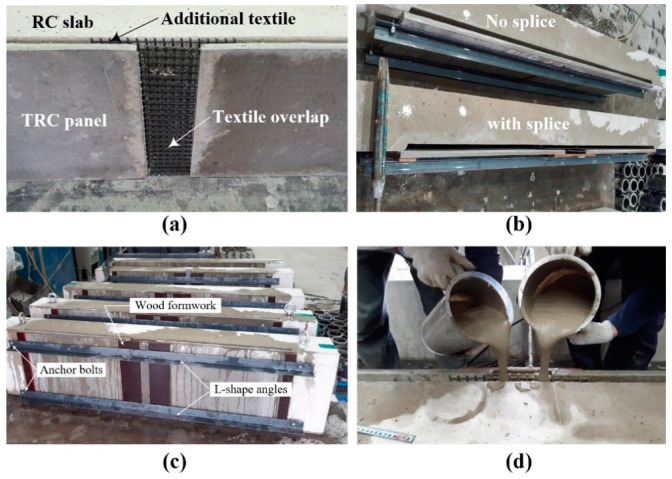
Strengthening process: (**a**) installation of TRC panels; (**b**) assembling of panels; (**c**) water spraying; and (**d**) grouting.

Figure 13 depicts the strengthening process of the S series specimens. The slab was set vertically and a set of the lap-spliced TRC panels was assembled with the bottom face of the RC slab (Figure 13a). It should be noted that the top face of the panel (rough surface) shown in Figure 12b faced the slab. A 300.0 mm-long plywood formwork was assembled over the lap-spliced zone, and the lap-spliced panels were fixed to the RC slab by a set of L-shape angles and anchor bolts (Figure 13c). The concrete slab and TRC panel were maintained in a water saturated state for 2 h. Figure 13d shows the grout filling process. The specimens were cured at a room temperature for 24 h, and then steam cured for 8 h.

### 3.4. Test Results of Strengthened Slabs and Discussion

#### 3.4.1. Setup and Instrumentation for Full-Scale Flexural Tests

**Figure 14:**
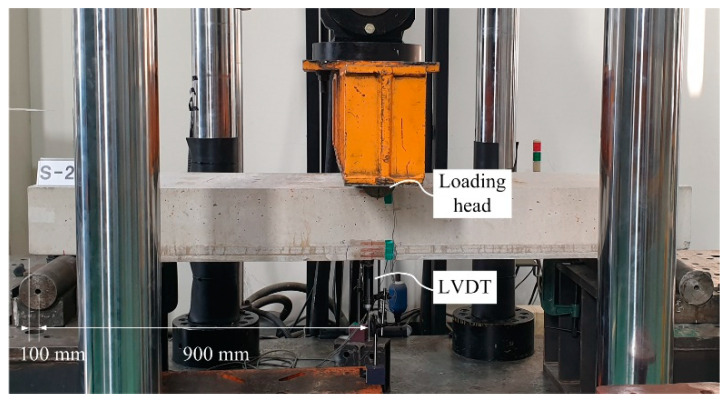
Full-scale flexural test setup and instrumentation.

The flexural performance of the strengthened slabs was assessed by a three-point flexural test using a universal testing machine with a capacity of 2000 kN (Daekyung Ltd. Co., Seoul, Korea) (Figure 14). The loading was applied through displacement control at a speed of 1 mm/min, and continued until the specimen did not show a significant post-yielding behavior after the peak load. Moreover, if a sudden collapse of the specimen due to extreme cracking or shear failure was anticipated visually, the loading was stopped for safety.

#### 3.4.2. Load-Displacement Behavior

**Figure 15:**
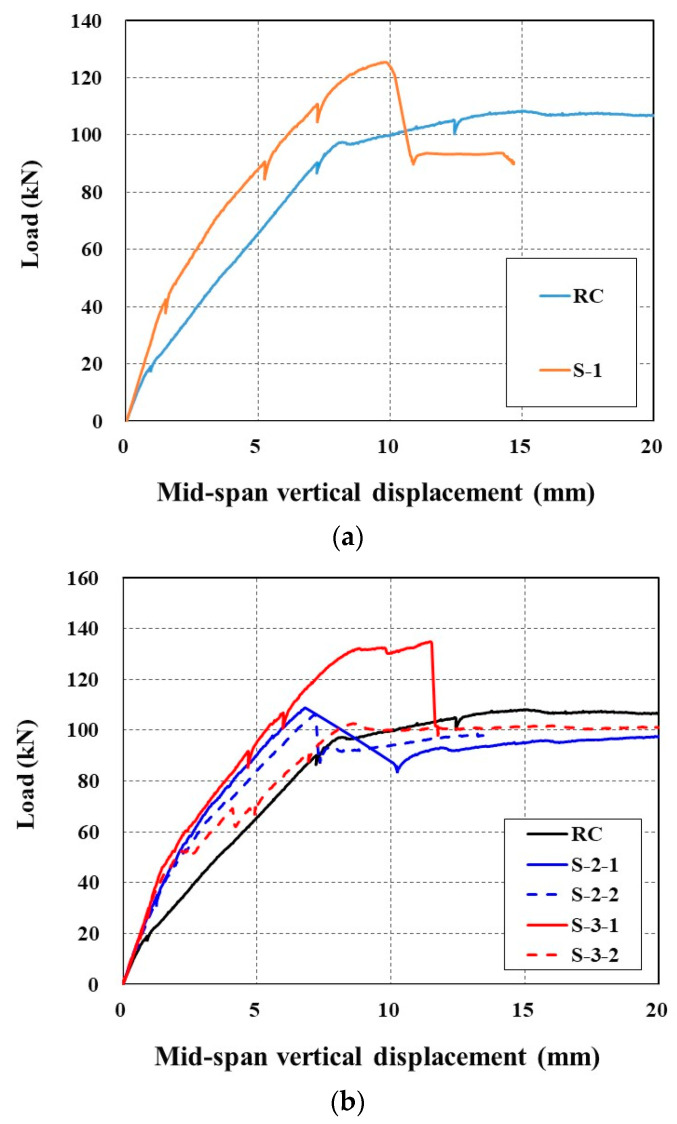
Comparison of load-displacement curves: (**a**) RC and S-1 specimens; and (**b**) RC, S-2, and S-3 specimens.

Figure 15 plots the applied load versus mid-span vertical displacement curves of the full-scale slab specimens tested under flexure. As shown in Figure 15a, the RC specimen (control) exhibits typical quasi-trilinear behavior, e.g., a linear stage until the initiation of cracks (load level = 19 kN), followed by a second stage resisting the load until yield of the steel reinforcement (load level = 82 kN), and finally, a third stage showing a plateau where only the displacement increases after yield of the steel reinforcement. Note that the cracking of concrete was identified by visual observation, but the yield of the steel reinforcement was evaluated by the measured strain through the strain gauge mounted on the bottom reinforcement (16 mm bars, Figure 9a).

The load-displacement response of the S-1 specimen without a lap splice indicated four-stage behavior. A linear behavior can be distinguished until the initiation of cracks in the first stage. In the second stage, the load is increased until the yield of steel reinforcement with two stress drops due to concrete cracking. The third stage shows an increase of the load due to the strengthening effect of the TRC panel, followed by abrupt failure in the fourth stage. The specimens strengthened with the TRC panel undergo smaller displacement than the RC specimen under the same load level. Note that sudden load drop after the peak load is due to the debonding of the TRC panel from the slab.

Sets of two specimens were fabricated and tested for each S-2 and S-3 series specimens with lap splice. The load-displacement responses of the S-2 series specimens until the peak load are similar to each other, and showed two stages; e.g., the first stage is linear until initiation of concrete cracks, and the second stage is approximately linear until abrupt failure.

On the other hand, quite different load-displacement responses were observed for the S-3 series specimens, and were inconsistent with each other. As mentioned in Section 3.1, the lap sliced joint of the S-3 series specimens was reinforced with an additional 300 mm-long textile grid within the grouting space. The S-3-1 specimen showed the largest stiffness and peak load among the strengthened specimens, even including the specimen without a lap splice. However, the S-3-2 specimen failed before the yield of the steel reinforcement by debonding of the TRC panel from the slab.

Table 7 summarises the failure test results of all sets of specimens. The steel reinforcement was assumed to have yielded when the value of the measured strain reached 0.002. Compared to the RC specimen (control), the initial cracking load caused yielding of the steel reinforcement, and the ultimate load-carrying capacity of the strengthened specimens improved by average values of 53%, 30% and 7%, respectively. Except for the S-3-2 specimen, the mid-span vertical displacement of the strengthened specimens at the load level that caused the steel yielding of the unstrengthened specimen was reduced by 27% on average.

#### 3.4.3. Cracking Patterns and Failure Modes

**Figure 16:**
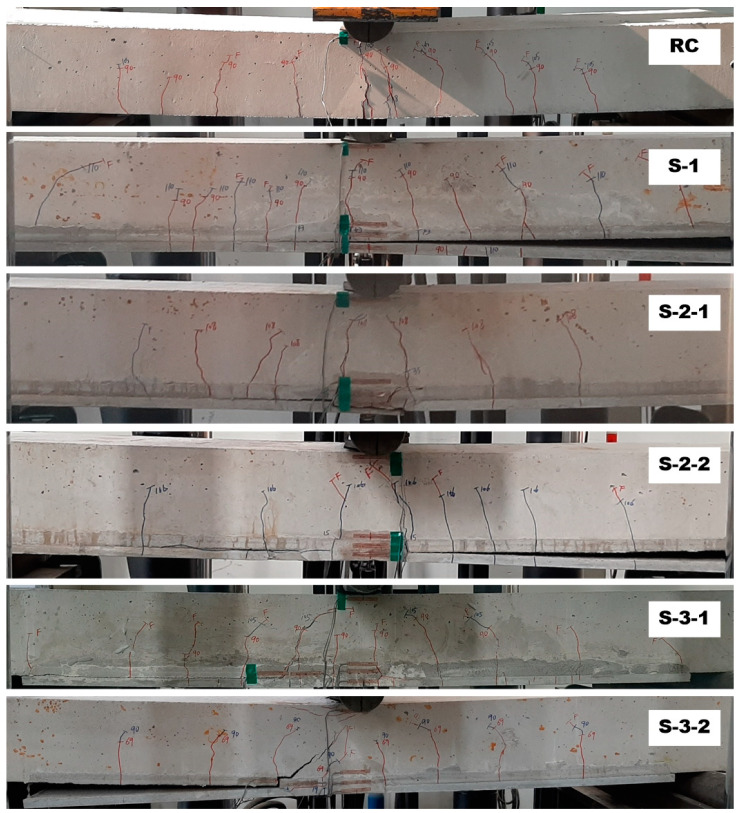
Side view of slab specimens after failure test.

Figure 16 illustrates the crack patterns of the specimens after the test. The RC specimen shows a typical flexural failure mode. Except for the S-2-1 specimen, all the strengthened specimens experienced flexural cracks, and finally failed by debonding at the interface between the TRC panel and grouting. The debonding of the TRC panel from the concrete slab is due to insufficient bond performance, and led to sudden failure. The debonding failure of strengthening materials from the concrete substrate is often observed by flexural tests for the externally bonded FRP system.

**Figure 17:**
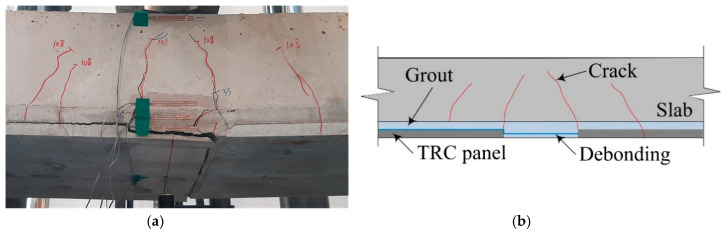
The S-2-1 specimen: (**a**) lap-spliced joint; and (**b**) failure mechanism.

Furthermore, the structural joint of the strengthened specimen (Figure 17) is quite complicated, physically as well as materially, where a RC slab (concrete) and two textiles lap-spliced TRC panels (mortar) are connected by bonding with CIP grout. As expected, the textile lap-spliced joint experienced cracks in an earlier loading step. Compared to the S-2 series specimens, smaller cracking width at the lap-spliced joint was observed for the S-3 series specimen. Therefore, the lap splice joint reinforced by the additional textile within the grout is considered to be effective.

#### 3.4.4. Discussion

Although the initial cracking load and the stiffness of the slab were somewhat increased by the strengthening with the TRC panel, the influence of the strengthening on the ultimate load carrying capacity of the slabs was insignificant. This is determined on the basis of five strengthened slabs out of six having failed, mainly by the debonding of the TRC panel from the slab. The debonding of the panel caused premature failure, and thus the TRC panel bonded to the slabs was unable to develop a sufficient composite action, and hence the strengthening effect. When an RC slab is designed for strengthening by the TRC panel with grout, the debonding failure is an undesirable failure mode, as it usually causes sudden catastrophic failure.

Furthermore, the influence of the lap-spliced of TRC panels on the strengthening, determining which was one of the objectives of this study, could not be investigated due to the premature bonding failure. Therefore, a viable grouting material must be developed for the proposed method of strengthening by a lap-spliced TRC panel in future work.

## 4. Flexural Behavior of Re-Strengthened Slabs

### 4.1. Re-Strengthening of Damaged RC Slabs

**Figure 18:**
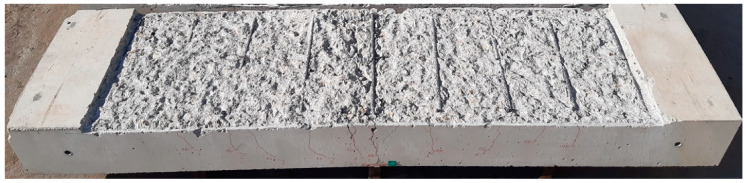
Damaged RC slab after removing strengthened section by chipping.

In this study, the failure-tested specimens (hereinafter damaged RC slabs) in Section 3.4 were re-strengthened by the same procedure described in Section 3.3. Note that the damaged RC slabs have more than one through thickness crack and permanent vertical displacement. The section strengthened with the TRC panel and grout of the damaged slabs was completely removed by concrete chipping (Figure 18). Furthermore, the tensile reinforcement of the damaged slabs completely yielded, and thus could no longer carry tensile stress. Therefore, the objective of the re-strengthening of the damaged RC slabs by the TRC panel and grout is to investigate whether the yielded steel reinforcement in the damaged slab can be replaced by a TRC panel.

**Figure 19:**
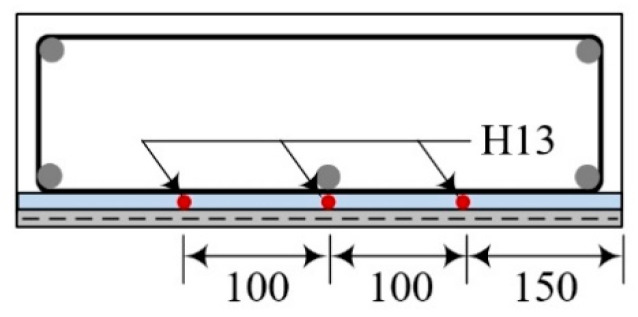
Additional tensile reinforcement (13 mm-bar) details (RS-2 series specimens).

Table 8 lists characteristics of full-scale slab specimens re-strengthened by the TRC panel and grout (hereinafter the re-strengthened slabs). The RS series denote the S series specimens in Section 3.4 that were re-strengthened with the TRC panel. The RRC and RS-1 specimens are re-strengthened with the full-length TRC panel, and RS-2 and RS-3 series specimens are strengthened with the lap-spliced TRC panel. The lap splice joint is located at the mid-span of the specimen. For the RS-2 series specimens, three additional H13 bars were arranged to partially replace the yielded reinforcement of the damaged slabs (Figure 19). Note that the lap splice joint of the RS-3 specimens was further reinforced with an additional 300 mm-long textile grid within the grouting space.

**Figure 20:**
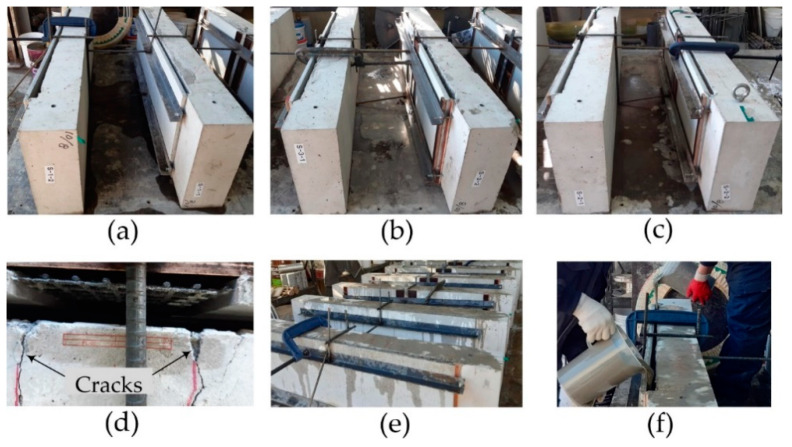
Re-strengthening process of the damaged slabs: (**a**) series RS-1; (**b**) series RS-2; (**c**) series RS-3; (**d**) lap splice joint; (**e**) water spraying; and (**f**) grouting.

Figure 20 illustrates the re-strengthening process of the damaged slab specimens. The re-strengthening and curing procedures are the same as those presented in Section 3.3. However, it should be noted that the bottom face of the panel shown in Figure 12b faced the slab. The reason why the bottom face of the panel was mounted to the slab is that the failure of the slabs strengthened with the top face of the panel (rough surface) was associated with debonding of the TRC panel at the interface between the panel and grout, as presented in Section 3.4.

### 4.2. Test Results of Re-Strengthened Slabs and Discussion

#### 4.2.1. Load-Displacement Behavior

**Figure 21:**
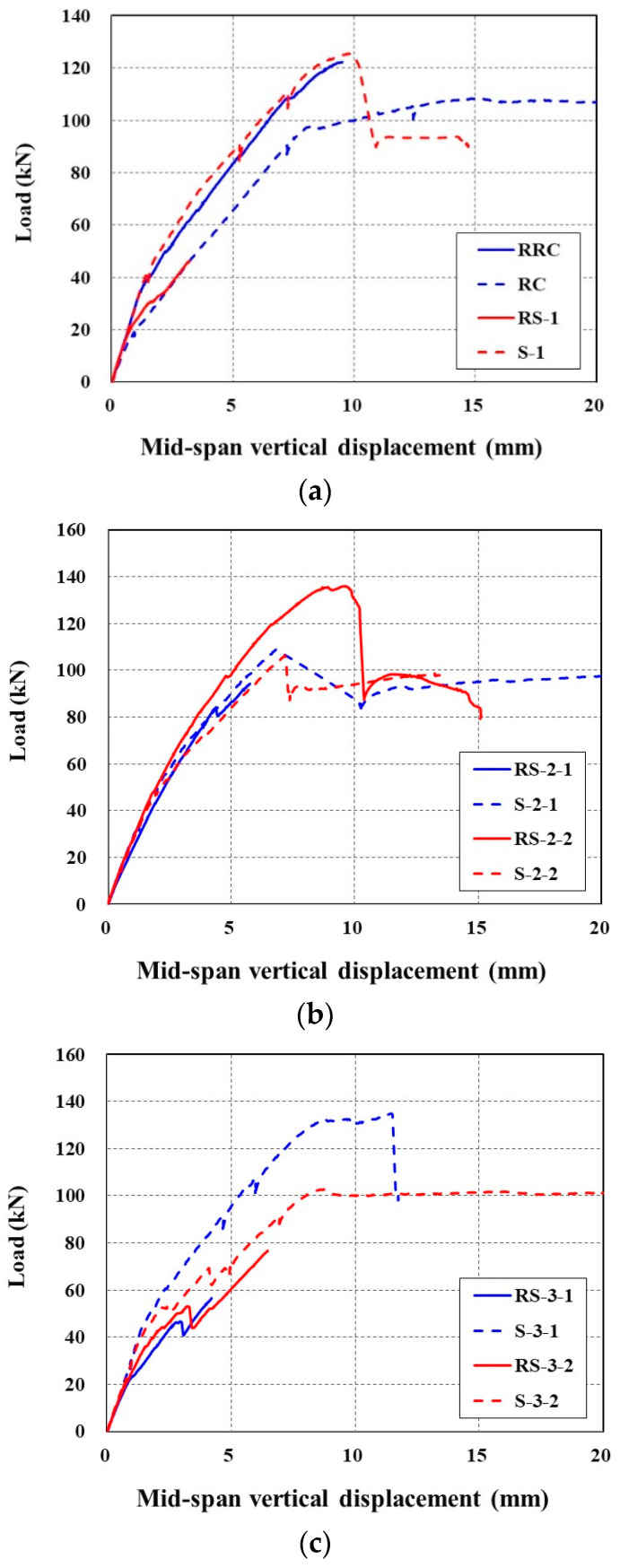
Comparison of load-displacement curves of strengthened specimens with re-strengthened specimens: (**a**) RRC and RS-1; (**b**) RS-2 series; and (**c**) RS-3 series.

Figure 21 presents the load versus mid-span vertical displacement curves of the re-strengthened specimens. Although the damaged slabs after the failure test, thus re-strengthened specimens, experienced a permanent displacement, the vertical displacements of the re-strengthened specimens measured during the test were initialised to zero in the figure.

The initial cracking load and the stiffness of the RRC specimen were significantly increased by re-strengthening. However, the RS-1 specimen was experienced re-strengthening effect due to composite action in an earlier loading stage of up to 20 kN, but failed by the delamination of TRC panel at 45 kN.

The load-displacement responses of the RS-2 series specimens with the additional steel reinforcement placed in the grouting space are almost identical to those of the (undamaged) strengthened specimens. On the other hand, the RS-3 series specimens experienced a re-strengthening effect in an earlier loading stage of up to 50 kN, but lost the strengthening effect at a load level of 50 kN to 75 kN. Note that sudden load drop of the RS-2 and RS-3 specimens after the peak load is due to the delamination of the TRC panel from the slab.

#### 4.2.2. Failure Modes

**Figure 22:**
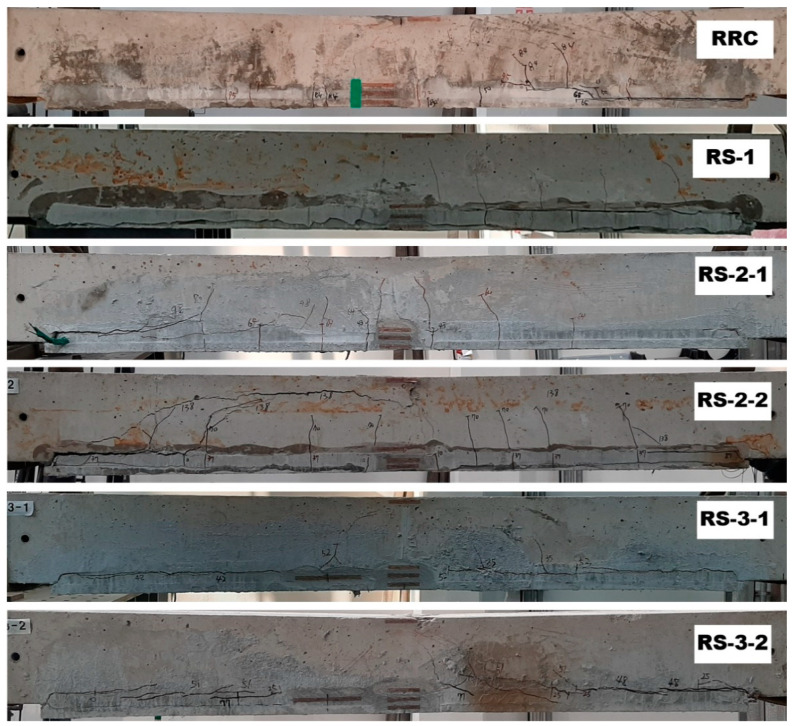
Failure mode of re-strengthening specimens.

Figure 22 presents the crack patterns and failure modes of the re-strengthened specimens after the test. The cracking patterns and failure modes of the re-strengthened specimens are quite different from those of the strengthened specimens. Except the RRC specimen, the failure of all other re-strengthened specimens was associated with the delamination and separation of the grouted section from the concrete substrate. The separation of concrete cover from the concrete substrate is usually observed for the flexural members strengthened by the externally bonded FRP system, if the bonding between the FRP system and the concrete substrate fails. Furthermore, no severe cracking or failure was observed at the lap-spliced joint.

For the RS-2 series specimens, the combined failure mode was observed with the occurrence of inclined tensile cracks after flexural cracking. The cracking of the re-strengthened specimens was changed from cracking induced by pure bending to cracking caused by the combined action of flexure and shear due to the additional steel reinforcement.

As presented in Section 4.1, the strengthened section of the damaged slabs was completely removed by concrete chipping, and consequently the outer surface of the slab was also damaged. Therefore, delamination can occur along the interface between the CIP grout and the concrete, rather than the panel and grout interface.

#### 4.2.3. Discussion

The results of the failure test for the re-strengthened slabs indicated that the damaged slabs can be re-strengthened by the TRC panel and grout. The test results clearly indicate that re-strengthening by the TRC panel with the additional steel reinforcement is more effective than that of the TRC panel only. Although the same grouting material was used for the fabrication of the re-strengthened specimens, debonding of the TRC panel and grout was not observed. One of the possible reasons may be that the bottom side of the panel, e.g., the precast panel surface facing the steel formwork during the cast, was grouted. Therefore, the most important factor for successful strengthening by the TRC panel and grout is to obtain composite action between the TRC panel and concrete substrate.

## 5. Conclusions and Future Study

This paper proposed an on-site connecting method for precast TRC panels by the lap splicing of textile grids. The tensile behavior of a TRC system with lap-spliced textile was experimentally investigated by a direct tensile test. Full-scale concrete slabs were strengthened by the lap-spliced TRC panel and CIP grout, and tested under flexure to validate the performance of the proposed connecting method. Moreover, the failure-tested specimens were re-strengthened by a new TRC panel system, and tested again under flexure to investigate whether the TRC panel can replace the yielded steel reinforcement. The following conclusions and recommendation for a future study can be drawn.

From the results of the direct tensile test conducted for the lap-spliced TRC system, the minimum lap splice length is considered to be 100 mm. The maximum value of the ultimate tensile strength of the lap-spliced TRC system is at most 78.6% of the non-lap-spliced case. The influence of matrix properties on the ultimate tensile strength of the lap-spliced TRC system is significant. Although the compressive strength of the mortar used is about 78% of that of the grout, for the same lap splice length, the ultimate tensile strength of the TRC system with mortar is almost two-fold greater than that of the TRC system with grout. Furthermore, the lap-spliced TRC specimens with grout failed by splitting, and finally delamination along the lap-spliced face of the textile.

Six full-scale RC slab specimens were strengthened by TRC panels with and without lap splice, and failure tested in flexure. The results of the failure test indicated that the initial cracking load and the stiffness of the slab were somewhat increased by the strengthening with the TRC panel. However, five strengthened specimens out of six failed mainly by the debonding of the TRC panel from the slab. The debonding of the panel caused premature failure, and thus the TRC panel bonded to the slabs was unable to develop a sufficient composite action, and thus strengthening effect.

The failure-tested specimens (damaged slabs) were re-strengthened by the TRC panel and grout to investigate whether the yielded steel reinforcement in the damaged slab can be replaced by a TRC panel. Although the same grouting material was used, the bottom face of the panel was mounted to the slab. The results of the failure test for the re-strengthened slabs indicated that the initial cracking load and the stiffness of the damaged slabs were significantly increased by re-strengthening. Furthermore, the failure of the re-strengthened specimens was mainly associated with the delamination and separation of the grouted section from the concrete substrate. Moreover, no severe cracking or failure was observed at the lap-spliced joint. Therefore, an on-site connecting method proposed in this study for precast TRC panels by lap splicing of textile grids can be considered as an effective method.

When dealing with externally bonding strengthening methods, including a strengthening method by the precast TRC panel with grout, the debonding failure is an undesirable failure mode, as it usually causes sudden catastrophic failure. Therefore, a viable grouting material needs to be developed for the proposed method of strengthening. This paper presents the experimental study only. However, as a bonding model between the concrete slab and the precast TRC panel is developed, the behavior of the structural elements strengthened with the precast TRC panel and the CIP grout can be analysed by numerical methods.

It should be noted that the numbers of strengthened and re-strengthened specimens tested in this study were very limited, in order to obtain reliable test results. Thus, an increased number of specimens needs to be considered for a test program to evaluate the strengthening performance of CIP TRC panel.

The proposed strengthening method can also be used to build or to strengthen RC protective structures subject to direct impact loads such as projectile firing. Recently, a testing method has been proposed for TRC under impact load [31]. Therefore, if the proposed strengthening method is to be employed in military applications, the evaluation of the material and structural performance of the CIP TRC panel under impact loadings needs to be conducted.

## Figures and Tables

**Table 1:** Material properties of carbon textile grid (suggested by the manufacturer).

Fiber	Resin	Cross-Sectional Area of Yarn (mm^2^)	Tensile Strength (MPa)	Elastic Modulus (GPa)
3200 tex ^1^	Epoxy	1.81	3300	220

^1^ tex = Grams per kilometer of yarn.

**Table 2:** Mixture composition of mortar for tensile test (KICT, unit: kg/m^3^).

Cement ^1^	GGBS ^2^	Sand ^3^	Water	Superplasticiser
466	466	1024	278	3

^1^ Type I Portland cement specified in ASTM C150 [30]. ^2^ GGBS = Granulated Blast-furnace Slag. ^3^ Grain sise = 0.1 mm~0.4 mm.

**Table 3:** Mix composition of ready-mixed concrete (unit: kg/m^3^).

Cement	Water	Fly Ash	GGBS	Sand	Coarse Aggregate ^1^	Superplasticiser
263	167	56	56	828	934	2.63

^1^ Maximum grain sise = 25 mm.

**Table 4:** Characteristics of tensile test specimens.

Specimen ID	Matrix Type	Lap Spliced	$L_{LS}$ (mm)	No. of Specimen
TM-0	Mortar	No	-	3
TM-75	Mortar	Yes	75	3
TM-100	Mortar	Yes	100	3
TM-150	Mortar	Yes	150	3
TG-0	Grout	No	-	3
TG-75	Grout	Yes	75	3
TG-150	Grout	Yes	150	3

**Table 5:** The results for direct tension tests (average values of three tests).

Specimen ID	$L_{LS}$ (mm)	$f_{cr}$ (MPa)	$f_{fu}$ (MPa)	$f_{fu}$ Gain (%)	$\varepsilon_{fu}$
TM-0	-	4.6	3370.2 (760.9)	100	1.9 (0.3)
TM-75	75	4.2	2233.9 (162.3)	66.3	1.2 (0.1)
TM-100	100	4.5	2986.7 (201.1)	88.6	2.1 (0.1)
TM-150	150	3.8	2849.7 (358.9)	84.6	1.6 (0.4)
TG-0	-	3.1	2833.1 (137.1)	100	2.2 (0.2)
TG-75	75	3.2	1570.2 (111.2)	55.4	1.0 (0.1)
TG-150	150	3.3	2228.4 (296.1)	78.6	1.6 (0.3)

Note: value in ( ) is a standard deviation of test data.

**Table 6:** Characteristic of full-scale specimens for flexural test.

Specimen ID	TRC Panel Strengthened	Lap Spliced	Additional Textile	No. of Specimens
RC	-	-	-	1
S-1	Yes	-	-	1
S-2	Yes	Yes	-	2
S-3	Yes	Yes	Yes	2

**Table 7:** Test results of failure test for strengthened slab specimens.

Specimen ID	Concrete Cracking		Steel Yielding		Failure		Failur Mode
	Load (kN)	Displacement (mm)	Load (kN)	Displacement (mm)	Load (kN)	Displacement (mm)	
RC	18.8	0.9	82.5	6.5	108.4	15.0	Flexure
S-1	42.6	1.5	112.0	7.5	125.4	9.8	Debonding
S-2-1	35.3	1.3	-	-	108.8	6.8	Joint fracture
S-2-2	16.1	0.6	99.3	6.4	106.1	7.2	Debonding
S-3-1	30.2	1.0	117.7	6.9	134.9	11.5	Debonding
S-3-2	20.3	0.7	101.2	13.9	102.9	8.7	Debonding

**Table 8:** Characteristic of re-strengthened slab specimens for flexural test.

Re-Strengthened Specimen ID	Damaged Specimen ID	Lap Splice	No. of Additional Steel Bars	No. of Additional Textile within Splice	No. of Specimens
RRC	RC	No	-	-	1
RS-1	S-1	No	-	-	1
RS-2	S-2	Yes	3	-	2
RS-3	S-3	Yes	-	1	2

## Data Availability

The data presented in this study are available on request from the corresponding author.
